# Improving adolescents’ knowledge about mental health and depression: a randomized experimental study of web-based information

**DOI:** 10.3389/fdgth.2025.1640366

**Published:** 2025-11-18

**Authors:** Maria W. H. Kloek, Carolin Zsigo, Regine Primbs, Lucia Iglhaut, Sara Kaubisch, Charlotte E. Piechaczek, Pia-Marie Keim, Lisa Feldmann, Gerd Schulte-Körne, Ellen Greimel

**Affiliations:** 1 Department of Child and Adolescent Psychiatry, Psychosomatics, and Psychotherapy, Hospital of the Ludwig-Maximilians University (LMU) Munich, Munich, Germany; 2 German Center for Mental Health (DZPG), Partner Site Munich-Augsburg, Munich, Germany

**Keywords:** adolescents, web-based information, online, knowledge, depression, mental health

## Abstract

Adolescents often lack adequate knowledge about mental health and available professional support, which hinders timely help-seeking. As many adolescents seek information online, providing reliable web-based resources may enhance their mental health literacy. This randomized experimental study (preregistered at ClinicalTrials.gov: NCT05300217) examined the effectiveness and reception of innovative online information designed to improve adolescents’ knowledge of depression and mental health. A total of 77 adolescents aged 12–18 years were randomly assigned to one of two conditions: (1) web-based information on depression or (2) web-based information on strategies to promote mental health. Both interventions were derived from the evidence-based website “ich bin alles” (English translation: “I am everything”). Primary outcomes were knowledge of depression and knowledge of strategies to promote mental health, assessed immediately after the intervention and at 2- and 4-week follow-ups. Secondary outcomes included perceived visual aesthetics, ease of use, utility, and enjoyment. Adolescents showed significant increases in knowledge of depression [*F* (3, 76.67) = 13.29, *p* < .001] and knowledge of strategies to promote mental health [*F* (3, 76.41) = 7.89, *p* <.001] over time, attributable to the assigned information. Participants also rated the website's visual aesthetics, ease of use, utility, and enjoyment positively. Age-appropriate, visually appealing web-based information effectively improved adolescents’ knowledge of depression and mental health. Freely accessible, evidence-based online resources represent a scalable strategy to strengthen mental health literacy among young people.

## Introduction

The estimated prevalence of mental disorders among young people aged 10–24 years in European countries ranges from 13% to 22%. Of all mental disorders, depressive and anxiety disorders are the most common and represent the greatest health burden in this age group (1). Evidence shows that only one-third of children and adolescents with a depressive or anxiety disorder seek treatment (2), despite the negative consequences associated with early-onset mental health problems. For example, depressive disorder in adolescence is associated with difficulties in social functioning, including high rates of school drop-out (3). It also increases the risk of substance abuse, suicide, and physical illnesses such as cardiovascular disease in adulthood (4–6).

One major reason adolescents do not seek professional help is limited mental health literacy, including insufficient knowledge about mental disorders and available professional support (7, 8). Mental health literacy is defined as the knowledge and beliefs about mental health that help individuals to promote their own or others' mental health (9). According to Jorm (10), mental health literacy encompasses the following: “Knowledge of how to prevent mental disorders, recognition of when a disorder is developing, knowledge of help-seeking options and treatment available, knowledge of effective self-help strategies for milder problems, and first aid skills to support others who are affected by mental health problems” (p. 231). Kutcher et al. (11) refined this definition by also considering individuals’ help-seeking intentions and attitudes (i.e., stigma) toward mental health disorders as part of mental health literacy.

Study findings from the systematic review by Singh et al. (12) support the notion that adolescents have limited mental health literacy. This review specifically examined adolescents' levels of mental health literacy in relation to depression, i.e., depression literacy. Most adolescents struggle to recognize depressive symptoms and prefer informal sources of help, such as friends or family, over professional support. The authors concluded that adequate depression literacy could enable adolescents to recognize depressive symptoms early and seek professional help. Improving depression literacy could therefore help adolescents recognize symptoms earlier and encourage help-seeking (10).

Most adolescents use mobile applications (apps), podcasts, websites, and social media platforms to quickly access new information and ideas (13). It is common for adolescents to turn to these web-based resources when exploring physical and mental health information. The health-related topics that young people aged 14–25 years search for the most include: fitness, nutrition, stress, depression, and anxiety (14, 15). When mental health problems emerge, young people report that they most commonly use the internet to seek information about their symptoms (16, 17). Adolescents generally initiate their search online using search engines (e.g., Google) to find mental health information (18). They value accessibility, anonymity, and low cost as key benefits when seeking health information online (14).

Despite increasing internet use among adolescents, few interventions have rigorously evaluated whether online mental health resources effectively improve knowledge or help-seeking intentions. Most existing research interventions that primarily aim to enhance mental health literacy through information dissemination have been conducted in school settings.

Several educational programs have demonstrated that school-based approaches can successfully improve adolescents' knowledge of depression and mental health. For example, studies in the U. S. and Australia have examined the effectiveness of classroom-based programs such as the *Adolescents Depression Awareness Program* (ADAP (19–21); and *Headstrong* (22). ADAP consisted of a three-hour curriculum including interactive lectures, videos, and group projects. *Headstrong* is a more extensive program, comprising ten hours of curriculum content delivered over a period of five to eight weeks. Adolescents who participated in these programs demonstrated increased knowledge of depression and stronger help-seeking intentions compared to those in the control group.

In contrast to school-based interventions, evidence on the effectiveness of web-based approaches aimed at improving adolescents' mental health literacy through information dissemination remains limited. Only two studies have evaluated the impact of providing online mental health information designed to enhance mental health literacy in adolescents (23) and young adults (24). Adolescents and young adults who received text materials on depression, anxiety and suicidality by e-mail showed an increase in help-seeking intentions (23, 24), and a decrease in depression stigma compared to the control condition (24). Change in knowledge was assessed as an outcome only in the study by Taylor-Rodgers and Batterham (24), who found an increase in knowledge about anxiety but not about depression. This scarcity of research highlights the need for further evaluation of scalable web-based resources designed to improve adolescents' mental health literacy.

One such resource is the innovative German website “ich bin alles” (https://www.ich-bin-alles.de; English translation “I am everything”), developed to improve mental health literacy among adolescents through engaging, evidence-based content. The website was developed by the Department of Child and Adolescent Psychiatry, Psychosomatics, and Psychotherapy at the Ludwig-Maximilians-University (LMU) University Hospital in Munich in collaboration with the “Beisheim Stiftung” and media partners. Its main purpose is to disseminate evidence-based information on depression and general mental health to adolescents and their families or carers (25). The mental health section provides information on strategies to promote general mental health (e.g., reducing stress, exercising, engaging in positive activities) and on options for seeking professional help. Other sections focus specifically on depression in adolescents, including its symptoms, etiology, course, and treatment options based on the German guideline for treating depressive disorders in children and adolescents (26).

Importantly, the website was created using a participatory design approach involving adolescents and professionals to ensure that both the content and visual design are age-appropriate and appealing. The platform uses multiple formats, including text, illustrations, animations, audio, and video, to accommodate different learning preferences. This combination of participatory design and multimedia formats represents a novel and engaging approach to promoting mental health literacy among adolescents.

This randomized experimental study examined the impact of two types of information from the “ich bin alles” website: (1) information on depression and (2) information on strategies to promote mental health. The main objective was to examine changes in two specific components of mental health literacy: knowledge of depression and knowledge of strategies to promote mental health. Participants' knowledge was assessed immediately before and after receiving (1) information on depression or (2) strategies to promote mental health and again at two- and four-week follow-ups. In addition, we evaluated adolescents` perceptions of the information in terms of its visual aesthetics, ease of use, utility, and enjoyment.

Based on previous findings from school-based educational programs (19–22), we hypothesized that participants would demonstrate an increase in knowledge after receiving the respective information. Specifically, we expected participants who received information about depression to demonstrate greater knowledge of depression compared to those who received information about strategies to promote mental health. Secondly, we expected participants who received information about strategies to promote mental health to demonstrate greater knowledge of these strategies than those who received information about depression. Given the participatory design and multimedia features of the website, we also expected adolescents to evaluate its usability and appeal positively.

## Materials and methods

### Participants

Study staff from the Department of Child and Adolescent Psychiatry, Psychosomatics, and Psychotherapy at the LMU Hospital in Munich recruited adolescents between January 2021 and May 2022. Recruitment strategies included flyers and two contact lists: one provided by the local authority and one comprising adolescents who had previously participated in studies at the department.

The study was approved by the ethics committee of the Medical Faculty of the LMU Munich (No. 20115). All participants were informed in detail about the study procedures and aims. Participants aged 18 years provided written informed consent; those under 18 years provided written assent, with additional consent from a parent or legal guardian.

Inclusion criteria were: age between 12 and 18 years, an intelligence quotient (IQ) ≥ 80, no current psychiatric diagnosis, no past diagnosis of depressive disorder according to ICD-10 (27), and sufficient German language skills to complete study materials. IQ was assessed using the *Culture Fair Intelligence Test – Revision* [CFT-20-R; (28)]. The absence of ICD-10 diagnoses of mental disorders was confirmed using a well-established diagnostic interview [*Kinder-DIPS*; Diagnostic Interview for Mental Disorders in Children and Adolescents; (29)].

A total of 77 participants met inclusion criteria and were randomized to either the *depression information group* (*n* = 39) or *mental health information group* (*n* = 38). According to an *a priori* power calculation, this sample size provided adequate statistical power to detect the expected effects (see Supplementary Material). Demographic characteristics of both groups were comparable and are shown in Table 1.

**Table 1:** Demographic characteristics of the participants.

Characteristics	Information on depression (*n* = 38)	Information on mental health (*n* = 39)	*p*
Age in years, mean (SD)	15.61 (1.67)	15.57 (1.92)	.912^a^
Age range in years	12–18	12–18	–
Sex, *n* (% female)	20 (53%)	20 (51%)	.906^b^
IQ, mean (SD)	109.54 (12.95)	108.31 (13.94)	.696^a^
Parental socioeconomic status, *n* (%)^c^			.264^b^
Lower	0	0	
Middle	6 (16%)	9 (24%)	
Upper	31 (84%)	29 (76%)	

a T-test.

b Chi-square test.

c We used the education level of both parents as indicator of parental socioeconomic status, which is classified as lower, middle, and upper (40), *N* = 75, 2 missing.

### Procedure

This single-blind randomized trial (ClinicalTrials.gov Identifier: NCT05300217) included two parallel experimental groups. Participants were blinded to group allocation, while study personnel involved in data collection were aware of assignments. All sessions were conducted by trained psychology students.

**Figure 1:**
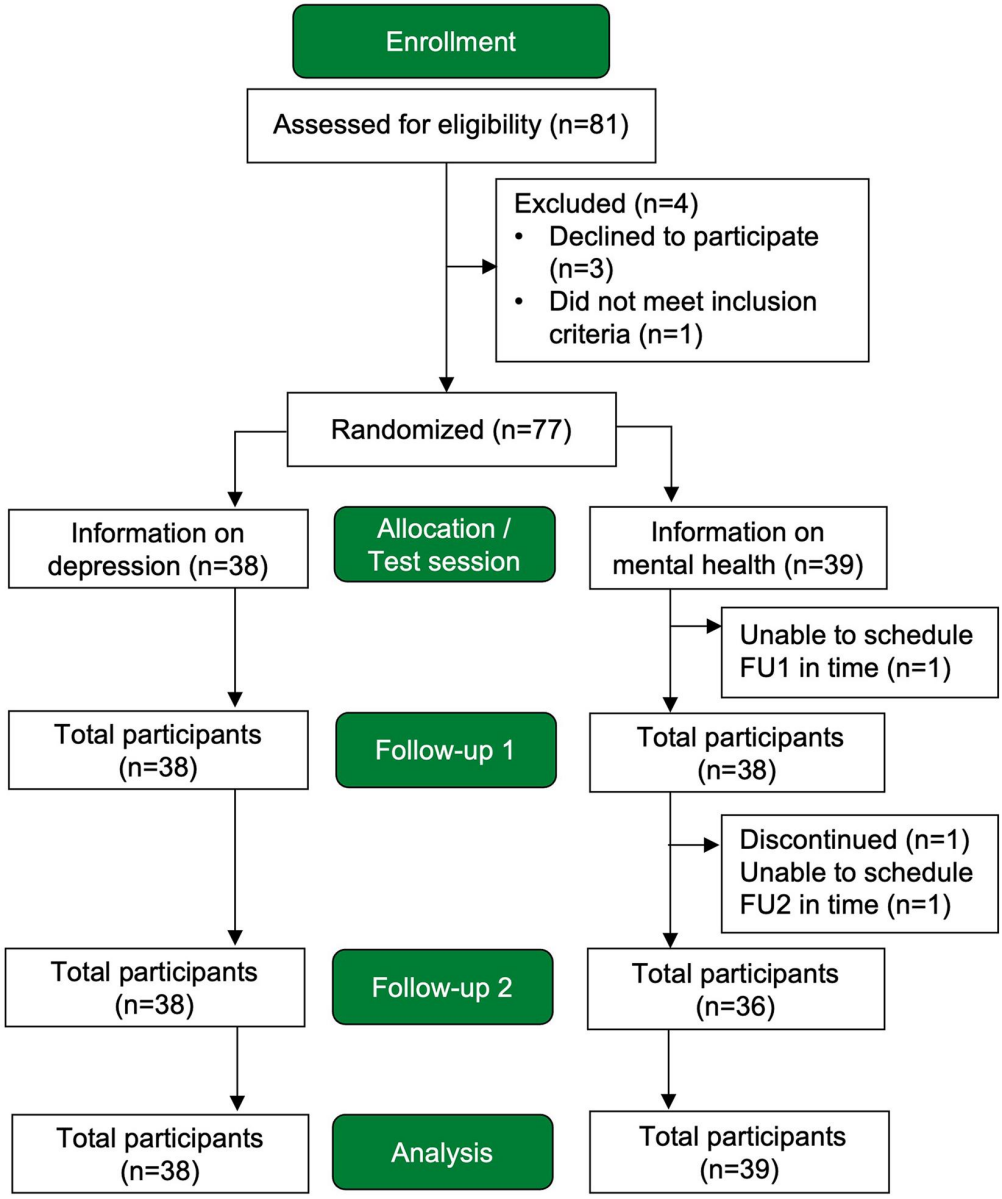
Flowchart showing the number of participants throughout the study.

Participants completed one test session (T1) and two follow-up (FU) appointments scheduled two weeks (FU1) and four weeks (FU2) after T1, respectively. Data collected outside these timeframes (FU1: after 2–3 weeks; FU2: after 4–5 weeks) were treated as missing. The flow of participants throughout the study, including dropouts and exclusions, is shown in Figure 1.

The T1 session lasted between two and three hours and comprised three parts: a pre-test (pre-T1), random allocation to the respective information, and a post-test (post-T1). The pre-test included a diagnostic assessment and an IQ test. Once eligibility had been confirmed, participants were randomized into two study groups using a computer-generated random number sequence. Randomization was stratified by age (<15 and ≥15 years) and sex, with an allocation ratio of 1:1. Participants were assigned to the intervention group by investigators (RP and LI), who were not involved in data collection. After the diagnostic assessment, participants completed the pre-test questionnaires and provided their demographic information.

The web-based information was subsequently presented (see below for details). Finally, participants completed the post-test questionnaires. The FU sessions consisted of participants completing the post-test questionnaires again and lasted approximately 30–45 min.

Sessions were primarily conducted at the facilities of the research department. Some experimental sessions had to be conducted online due to restrictions caused by the COVID-19 pandemic during the evaluation period. Online sessions were conducted via screen sharing using RED connect (RED Medical Systems GmbH). Each participant visited the research department at least once for a face-to-face test session.

### Presentation of the web-based information

An offline version of the website “ich bin alles” was used for the study. It contained only the materials relevant to the two intervention conditions: information on depression and information on strategies to promote mental health. Both were similar in structure and design but covered distinct, non-overlapping content areas. The public version of the website was launched in September 2021, while data collection was still ongoing. No participant reported prior exposure to the website “ich bin alles” at any measurement point.

Before the presentation of the web-based information, participants in both groups were told that they would examine material from a website and were asked to review the content attentively. Study personnel notified them when the predefined time window had elapsed. Depending on the length of the text, podcast, or video, participants were given between two and eight minutes. During the presentation, study personnel remained with each participant to ensure compliance.

Participants in the depression information group viewed content explaining depression, including its frequency, symptoms, and course. This material also addressed contributing factors and sources of professional help. Participants in the mental health information group viewed content focusing on strategies to promote mental health. Topics included self-care, exercise and sleep management, positive thinking, stress management, and problem solving.

### Outcome measures

**Figure 2:**
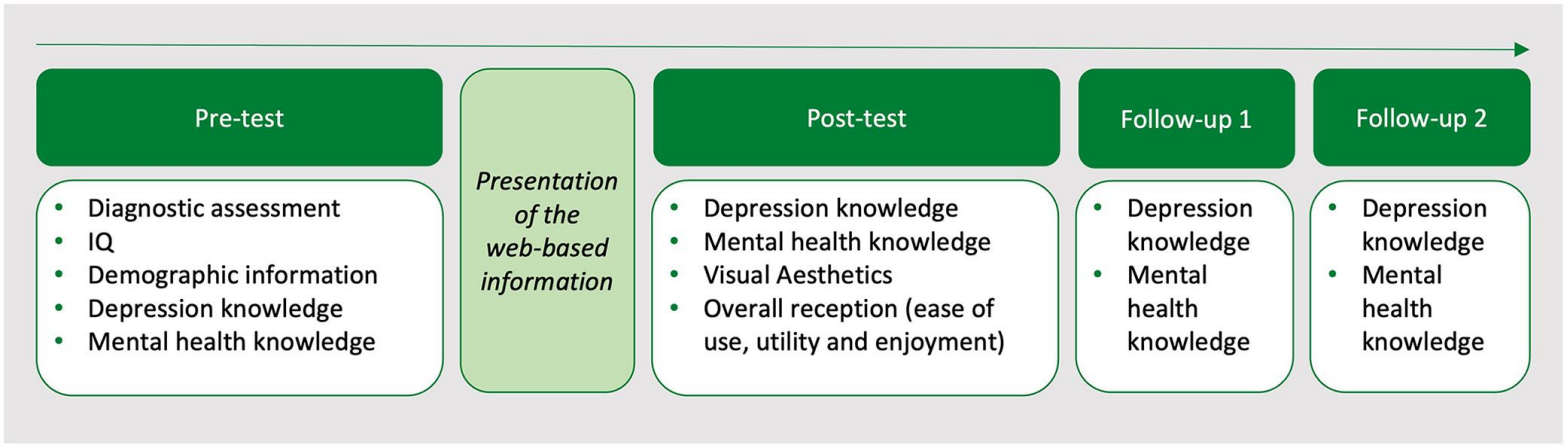
Overview of the outcome measures at the study measurement points.

An overview of all outcome measures applied at each time point is provided in Figure 2, and detailed descriptions are summarized in Supplementary Table S1.

#### Primary outcome measures

The primary outcomes were two knowledge components of mental health literacy, reflecting the content of the website “ich bin alles”: (1) knowledge about depression and (2) knowledge about strategies to promote mental health. As no validated questionnaire covering the website's content was available, two self-report questionnaires were developed specifically for this study based on the website's informational materials. Items were derived directly from the website's informational materials and formulated in consultation with mental health professionals. Both questionnaires were pre-tested with adolescents to ensure clarity and age-appropriate language, and items were revised accordingly. Each questionnaire comprised 27 items (9 true/false and 18 multiple-choice) with one point awarded per correct answer (range: 0–27); higher scores indicated greater knowledge. Internal consistency at post-test was acceptable to good (39) (*α* = .70 for depression knowledge and *α* = .85 for mental health knowledge).

#### Secondary outcome measures

The secondary outcome measure was the overall reception of the website, including *visual aesthetics, ease of use*, *utility*, and *enjoyment* of the web-based information. Visual aesthetics were assessed using the *Visual Aesthetics of Websites Inventory – Short Version* [VisAWI-S; (30)], which consists of four items rated on a 7-point Likert scale. Higher scores indicate more positive evaluations, with mean scores above 4.5 reflecting a positive assessment of the website's visual layout.

Ease of use, utility, and enjoyment were measured using an eight-item questionnaire developed by the research group, with items rated on four response categories (strongly disagree, disagree, agree, and strongly agree), followed by a global rating using the German school grading system (range: 1 = excellent, 6 = insufficient).

### Statistical analysis

For both the mental health knowledge and depression knowledge questionnaires, raw scores were converted into index scores by dividing the total score obtained by the maximum possible score, and multiplying the result by 100. The index score thus represents the percentage of correct responses on each questionnaire. For the calculation of these indices, mean imputation was used for single missing items; this was the case for 0.29% of items on the depression knowledge questionnaire, and for none of the items on the mental health knowledge questionnaire.

All analyses were performed using IBM SPSS Statistics 29. To examine changes over time, mixed-model analyses of variance (ANOVAs) were employed due to their suitability for longitudinal data. Unlike standard repeated-measures ANOVAs, mixed models (also known as multilevel models) use all available data and do not exclude participants with missing values due to attrition (31). Thus, participants who provided data for at least one measurement point were included in the analyses, and missing data at later measurement occasions were handled using maximum likelihood estimation.

Change in knowledge of depression and in knowledge of strategies to promote mental health over time were analyzed using mixed-model ANOVAs with the factors group (depression information, mental health information) and time (pre-T1, post-T1, FU1, FU2).

Following the ANOVA omnibus tests, and to minimize multiple testing, we examined only specific contrasts of interest: (1) differences between the two groups at each measurement point and (2) differences between the first measurement point (pre-T1) and all subsequent measurement points within each group, to quantify short- and long-term effects.

To facilitate interpretation of the mixed model parameters for these contrasts as effect sizes, the dependent variables were standardized to z-scores (mean = 0, SD = 1), and group and time were represented by binary indicator variables. Standardized regression coefficients were preferred over traditional effect size indices such as *η*^2^ or Cohen's *d*, as these indices are not straightforward to compute in mixed models where variance components and sample sizes can differ across levels and time points. By standardizing the dependent variables, the resulting regression coefficients express changes in standard deviations units, allowing them to be interpreted using Cohen's conventions (0.2 = small effect, 0.5 = medium effect, 0.8 = large effect (32, 33).

Descriptive statistics were used to summarize perceived visual aesthetics and the overall reception of the website. For the eight items assessing ease of use, utility, and enjoyment, results were reported as the percentage of participants selecting each agreement category. The VisAWI-S and the global school-grade rating were analyzed as mean values. Differences between the two groups were tested using independent-samples *t*-tests and chi-square tests.

## Results

### Primary outcomes

**Figure 3:**
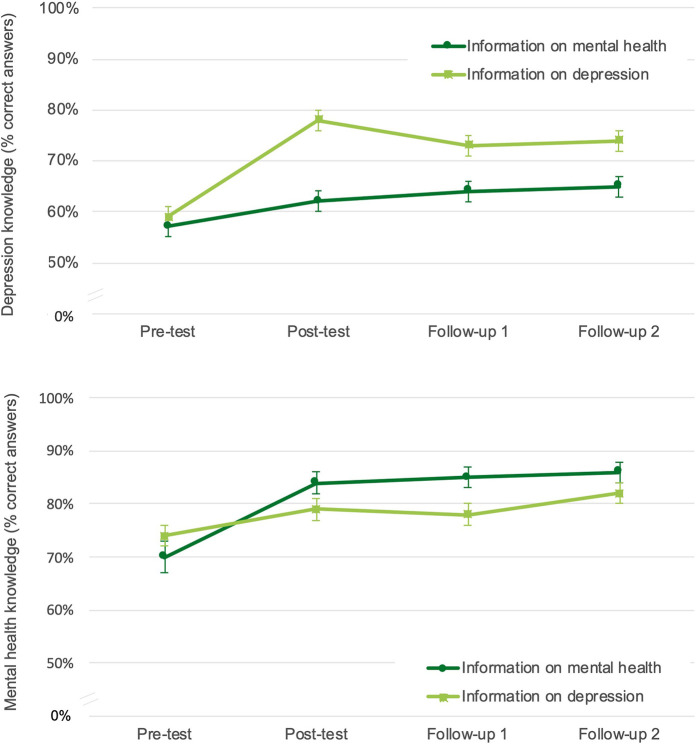
Changes in knowledge scores over time.

For changes in knowledge of depression, the mixed-model ANOVA revealed a significant main effect of group, *F* (1, 77.21) = 11.01, *p* = .001, a significant main effect of time, *F* (3, 76.67) = 38.82, *p* < .001, and a significant group × time interaction *F* (3, 76.67) = 13.29, *p* < .001. Means and standard deviations for this outcome are provided in Supplementary Supplementary Table S2. Figure 3 displays changes in knowledge of depression over time for both groups.

Pairwise contrasts and effect sizes are shown in Table 2. The groups did not differ significantly in knowledge of depression at pre-T1 (baseline) but differed significantly at post-T1, FU1, and FU2, with the depression information group showing higher knowledge scores. In both groups, knowledge of depression increased significantly from pre- to post-T1 and remained elevated at FU1 and FU2. These increases corresponded to medium effect sizes for the mental health information group and large effect sizes for the depression information group.

**Table 2:** Standard regression coefficients for pairwise contrasts on knowledge of depression.

Pairwise contrast	Standardized estimate (*β*)	Std. error	df	*t*	*p*	95% CI
Between groups (depression information vs. mental health information)						
At pre-test	0.11	0.22	77.00	0.48	.63	[−0.33; 0.54]
At post-test	1.04	0.19	77.00	5.51	**<**.**001**	[0.66; 1.41]
At follow-up 1	0.60	0.20	77.12	2.94	.**004**	[0.19; 1.00]
At follow-up 2	0.58	0.19	77.31	3.10	.**003**	[0.20; 0.95]
Within depression information group^a^						
Pre-test vs. post-test	1.26	0.11	77.00	11.44	**<**.**001**	[1.03; 1.47]
Pre-test vs. follow-up 1	0.96	0.14	76.35	7.07	**<**.**001**	[0.69; 1.23]
Pre-test vs. follow-up 2	1.03	0.12	75.96	8.63	**<**.**001**	[0.79; 1.27]
Within mental health information group^b^						
Pre-test vs. post-test	0.33	0.11	77.00	3.00	.**004**	[0.10; 0.54]
Pre-test vs. follow-up 1	0.47	0.14	77.24	3.48	**<**.**001**	[0.20; 0.74]
Pre-Test vs. follow-up 2	0.56	0.12	77.79	4.67	**<**.**001**	[0.32; 0.79]

Bold values indicate statistically significant results (*p* < .05).

a Depression information group received information on depression.

b Mental health information group received information on strategies to promote mental health.

For changes in knowledge of strategies to promote mental health, the mixed-model ANOVA revealed a significant main effect of time, *F* (3, 76.41) = 34.45, *p* < .001, and no significant main effect of group, *F* (1, 77.07) = 0.85, *p* = .36. However, there was a significant group × time interaction, *F* (3, 76.41) = 7.89, *p* < .001. Means and standard deviations for this outcome are presented in Supplementary Supplementary Table S2. Figure 3 displays changes in knowledge of strategies to promote mental health over time for both groups.

Pairwise contrasts and effect sizes are shown in Table 3. The groups did not differ significantly in knowledge at any measurement point (pre-T1, post-T1, FU1, and FU2). Both groups showed a significant increase in knowledge of strategies to promote mental health from pre- to post-T1, which remained elevated at FU1 and FU2. These increases corresponded to large effect sizes in the mental health information group and, by contrast, to small to medium effect sizes in the depression information group.

**Table 3:** Standard regression coefficients for pairwise contrasts on knowledge of strategies to promote mental health.

Pairwise contrast	Standardized estimate (β)	Std. error	df	*t*	*p*	95% CI
Between groups (mental health information vs. depression information)						
At pre-test	0.24	0.23	77.00	1.05	.30	[−0.21; 0.69]
At post-test	0.32	0.21	77.00	1.53	.13	[−0.74; 0.09]
At follow-up 1	0.42	0.22	77.09	1.93	.06	[−0.84; 0.01]
At follow-up 2	0.24	0.21	77.34	1.14	.26	[−0.65; 0.17]
Within mental health information group^a^						
Pre-test vs. post-test	0.85	0.10	77.00	8.51	**<**.**001**	[0.64; 1.04]
Pre-test vs. follow-up 1	0.91	0.10	77.71	9.25	**<**.**001**	[0.71; 1.10]
Pre-test vs. follow-up 2	0.98	0.11	77.05	9.05	**<**.**001**	[0.76; 1.19]
Within depression information group^b^						
Pre-test vs. post-test	0.29	0.10	77.00	2.83	.**01**	[0.08; 0.48]
Pre-test vs. follow-up 1	0.26	0.10	76.72	2.58	.**01**	[0.05; 0.45]
Pre-Test vs. follow-up 2	0.50	0.11	75.18	4.65	**<**.**001**	[0.28; 0.71]

Bold values indicate statistically significant results (*p* < .05).

a Mental health information group received information on strategies to promote mental health.

b Depression information group received information on depression.

### Secondary outcomes

#### Visual aesthetics

The perceived visual aesthetics of the website, as measured by the VisAWI-S index score, did not differ significantly between the two groups (*p* = .43). Both groups rated the web-based information very positively (VisAWI-S index > 4.5), with a mean rating of 5.97 (SD = 0.67) for the depression information group and 5.80 (SD = 1.12) for the mental health information group.

#### Ease of use, utility, and enjoyment

**Figure 4:**
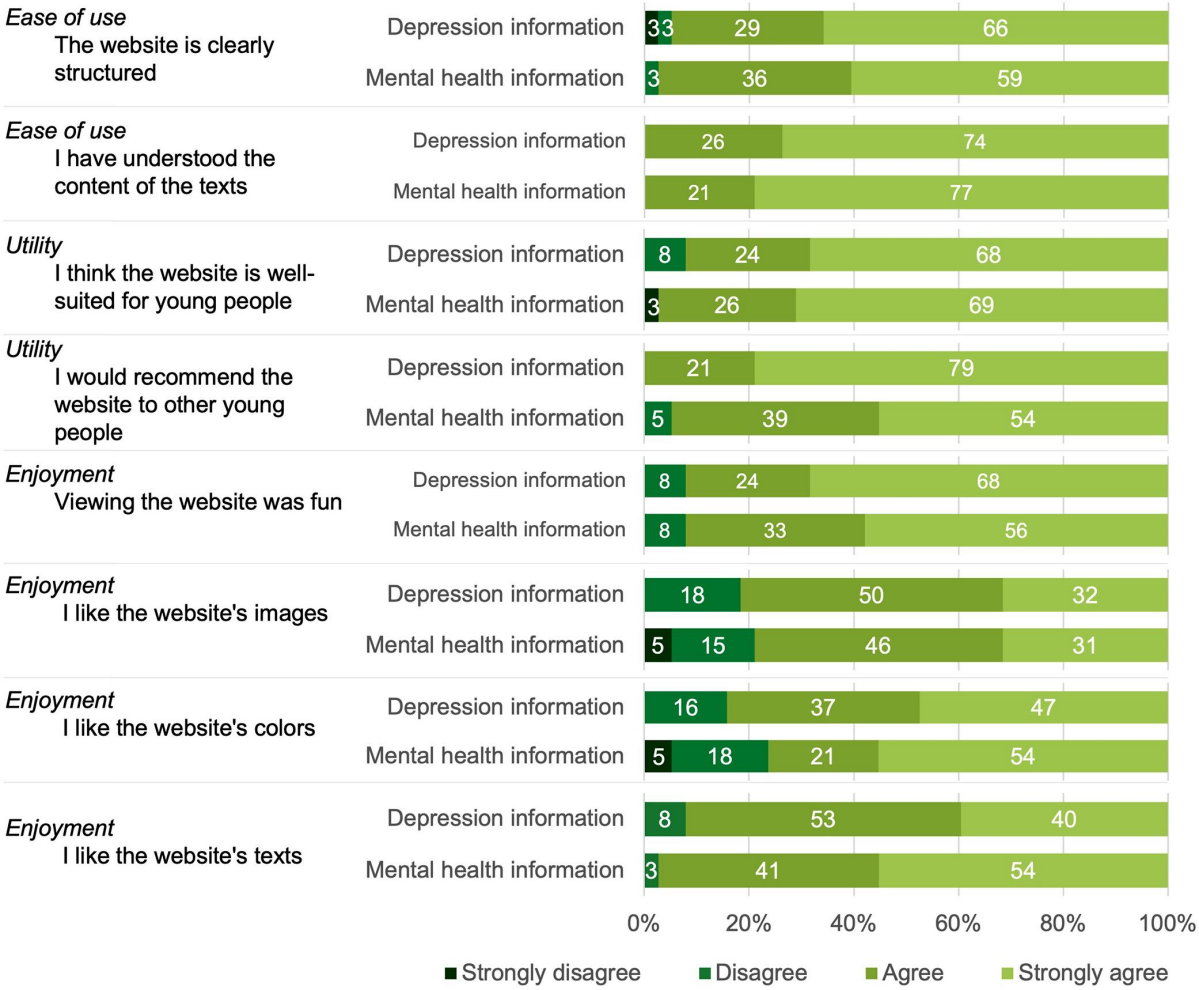
Participants’ perceived ease of use, utility, and enjoyment of the web-based information.

An overview of the descriptive results for the perceived ease of use, utility, and enjoyment of the website is shown in Figure 4. Overall, the website was received favorably by participants. There were no significant differences between groups after correction for multiple testing (all Bonferroni corrected *p* > .14).

Both groups assigned the website a school grade between 1 (“excellent”) and 2 (“good”). The mental health information group gave an average grade of 1.74 (SD = 0.61), and the depression information group gave an average grade of 1.58 (SD = 0.55). This difference was not statistically significant (*p* = .22).

## Discussion

This is the first randomized experimental study evaluating an adolescent-targeted, evidence-based website designed to enhance mental health literacy. Specifically, we examined whether exposure to information materials from the website “ich bin alles” increased adolescents' knowledge of depression and of strategies to promote mental health.

Three main findings emerged. First, receiving web-based information about depression or strategies to promote mental health led to significant increases in adolescents' knowledge in the respective topic area. Second, this newly acquired knowledge was largely retained two and four weeks after exposure Third, most participants rated both the visual design and the content of the website positively.

Our results align with prior evidence from school-based interventions such as the *Adolescent Depression Awareness Program* (ADAP), which also reported substantial knowledge improvements following brief educational sessions (19–21). Importantly, our study extends this literature by demonstrating that comparable outcomes can also be achieved through a web-based delivery format using multimedia materials, indicating that this approach can effectively improve adolescents' mental health literacy.

Consistent with our study hypotheses, the depression information group, compared with the mental health information group, showed a more pronounced increase in knowledge about depression. This suggests that adolescents who received depression-specific informational materials had a particular advantage in knowledge acquisition compared with those who viewed general mental health information. The maintenance of these gains over time further indicates that adolescents were able to successfully retain recently acquired knowledge about depression.

For knowledge of strategies to promote mental health, both groups improved over time, with no between-group differences at any measurement point. This finding suggests that all participants acquired a comparable understanding of strategies that support mental health. One possible explanation is that the depression information group also received some information about protective factors within the material on the causes of depression (e.g., taking the initiative to maintain control over one's situation). Participants in this group may have transferred their acquired knowledge about depression to a broader context of mental health. Consequently, their knowledge of strategies to promote mental health (e.g., stress management and self-care) may have improved sufficiently to eliminate between-group differences.

Participants' positive evaluations of the website “ich bin alles” in terms of ease of use, enjoyment, utility, and visual aesthetics further highlight its acceptability among adolescents. These results suggests that the web-based information was accessible, appealing, and well-tailored to adolescents' needs. This aligns with findings by Dysthe et al. (34), who analyzed 870 online questions and comments posted by adolescents about their experiences of depressive symptoms. The most frequently mentioned topics were self-management (“What can I do myself?”), etiology (“Why is it like this?”), and therapy (“What kind of therapy?”) The fact that the website “ich bin alles” addresses these topics indicates that its content closely aligns with the issues adolescents consider most relevant when seeking information about depression.

Our findings suggest that web-based dissemination can complement traditional school-based programs by offering scalable, user-driven education that enhances adolescents' mental health literacy beyond formal settings. The WHO Global Accelerated Action for the Health of Adolescents [AA-HA!; (35)] framework explicitly calls for innovative, scalable, and youth-centered approaches to improve adolescent health and well-being. In line with this strategy, the web-based approach of “ich bin alles” exemplifies an online, youth-tailored, evidence-based, and scalable educational resource that empowers adolescents to access mental health information.

### Strengths, limitations, future directions

One of the main strengths of this study relates to the inclusion of two experimental study groups which served as active control conditions for the respective primary knowledge outcomes. This design enabled a direct comparison of the effectiveness of different web-based information materials. In addition, the controlled experimental approach with a standardized test session represents a further strength, as it minimized variability between participants and thereby increased the likelihood of observing true effects attributable to the specific informational content. This enhances the internal validity of the findings.

Another advantage of this study is the scientific evaluation of a publicly available website. Users of the website “ich bin alles” have access to high-quality, evidence-based mental health information, with transparency regarding how the content is developed and by whom. Free access ensures that a broad range of adolescents can benefit from scientifically evaluated mental health information in a format tailored to their specific preferences and needs.

While the controlled experimental setting strengthened internal validity, it may have reduced ecological validity, as it differs from naturalistic website use, where adolescents access the material independently. In everyday contexts, engagement and learning may be influenced by factors such as attention, motivation, or competing online activities. To address this, users' experiences in the context of “ich bin alles” are currently being examined in a naturalistic study (ClinicalTrials.gov: NCT06668701), which will provide complementary insights into the website's benefits across different usage contexts. Future research could also integrate digital usage analytics, such as time on page, navigation patterns, or interaction metrics, to better understand how adolescents engage with web-based mental health information.

A further limitation concerns the study sample, which consisted of adolescents with slightly above-average IQ and predominantly higher socioeconomic status. These characteristics may limit generalizability, as lower parental socioeconomic status and below-average IQ among adolescents have been linked to limitations in knowledge acquisition (36, 37). Adolescents from lower socioeconomic backgrounds might also rate the overall reception of the website in a distinct way. For example, perceived utility (i.e., relevance of information) may depend on how well the topics align with the needs and experiences of less privileged groups. Future studies should therefore aim to recruit more diverse samples in terms of both socioeconomic status and IQ to explicitly test potential influences of these variables. Furthermore, gender differences in the use and perception of the web-based information were not examined and should be addressed in future research.

Another limitation relates to the use of self-developed outcome measures, for which external validity has not been evaluated. While this is a methodological limitation, the use of tailored instruments was necessary given the specific content of the website “ich bin alles”. Nevertheless, their content-based development, expert consultation, and acceptable internal consistency provide preliminary support for their suitability in assessing knowledge within the context of this intervention.

An important point to note is that the present study focused exclusively on the knowledge component of mental health literacy, while other components such as stigma and help-seeking efficacy were not assessed. This focus was chosen because knowledge represents a foundational element of mental health literacy and a prerequisite for reducing stigma and enhancing help-seeking behavior (11, 38). Given the extensive nature of the website's content, the questionnaires needed to be sufficiently comprehensive to capture the informational material. Including additional measures would have substantially increased the length of the assessment and may have increased the risk of response fatigue and measurement bias. Notwithstanding these considerations, future studies are encouraged to extend this research by incorporating measures of stigma and help-seeking efficacy to examine whether increases in knowledge translate into attitudinal and behavioral changes among participants.

### Practical implications

The results of our study demonstrate that age-appropriate web-based information presented in an appealing visual design can effectively increase knowledge about mental health and depression in adolescents. As most adolescents seek mental health information online (14, 16, 17) these findings highlight the importance of publicly available, evidence-based, and high-quality web-based resources on these topics.

A web-based dissemination approach has considerable potential to provide free, trustworthy information to adolescents, thereby helping to address the general lack of mental health knowledge in this age group. In the long term, high-quality, freely accessible web-based resources could enhance mental health literacy at the community level, which could facilitate earlier recognition of problems and encourage help-seeking, thereby supporting the prevention of mental health disorders. Integrating validated web-based tools into school mental health curricula or national youth portals could broaden their reach and support early intervention in youth.

## Data Availability

The datasets presented in this article are not readily available because the dataset generated and analyzed during the current study is not publicly available due to the confidentiality of participant information. De-identified aggregated data can, however, be made available upon request. Requests to access the datasets should be directed to Ellen Greimel, Ellen.Greimel@med.uni-muenchen.de.
