# Supplementary material for: Improving adolescents’ knowledge about mental health and depression: a randomized experimental study of web-based information
Source: Front Digit Health. 2025 Nov 18;7:1640366. doi: 10.3389/fdgth.2025.1640366 (PMC12669095; doi:10.3389/fdgth.2025.1640366)
Supplement: Supplementary file 1 [file Datasheet1.pdf]

Supplementary Material

Table S1. Overview of outcome measures used in the study

Measure	Construct	Example Item	Scale	Items	Scoring	Reliability (α)
Depression Knowledge Questionnaire	Knowledge about depression; developed by the research group based on website content	"If many close relatives have suffered from depression, it means that a child or adolescent will definitely develop depression as well."	9 True/False and 18 multiple-choice	27	Scores calculated as the sum of correct answers (range: 0–27); higher scores indicate greater knowledge	.70
Mental Health Knowledge Questionnaire	Knowledge about strategies to promote mental health; developed by the research group based on website content	"Replacing negative thoughts with positive ones can help improve mood."	9 True/False and 18 multiple-choice	27	Scores calculated as the sum of correct answers (range: 0–27); higher scores indicate greater knowledge	.85
Visual Aesthetics of Websites Inventory – Short Version (VisAWI-S)	Perceived visual aesthetics of the website; standardized measure	"Everything on the website fits together."	7-point Likert scale (strongly disagree to strongly agree)	4	Mean item score calculated; higher scores indicate more positive evaluations; with values > 4.5 reflect a favorable assessment of the visual layout	.88 (Thielsch & Moshagen, 2015
Website Reception Questionnaire – Ease of Use, Utility, and Enjoyment	Perceived ease of use, utility, and enjoyment of the website; developed by the research group	"I would recommend the website to other young people."	4 response categories (strongly disagree, disagree, agree and strongly agree)	8	Each item rated categorically on four response options; higher response categories indicating more positive evaluations	Not applicable

Website Reception Questionnaire – Global Rating	Overall reception of the website; developed by the research group	"What school grade would you give the website?"	German school grading system (1, 2, 3, 4, 5 or 6)	1	Lower scores indicate better evaluations; (1 = "excellent," 2 = "good," 3 = "satisfactory," 4 = "sufficient," 5 = "poor," and 6 = "insufficient")	Not applicable

Note. All outcome measures were self-report.

Table S2. Means and standard deviations for primary outcome measures for each group across all measurement time points

Primary Outcome Measures	Information on depression (n = 38)	Information on mental health (n = 39)
Depression knowledge (% correct answers)		
Pre-test, mean (SD)	59.01 (1.52)	57.44 (14.37)
Post-test, mean (SD)	77.55 (12.04)	62.23 (12.67)
Follow-up 1, mean (SD)	73.20 (15.34)	64.34 (10.87)
Follow-up 2, mean (SD)	74.27 (12.79)	65.39 (11.88)
Mental health knowledge (% correct answers)		
Pre-test, mean (SD)	74.14 (12.79)	70.31 (16.44)
Post-test, mean (SD)	78.72 (16.26)	83.91 (13.75)
Follow-up 1, mean (SD)	78.23 (16.88)	84.63 (13.80)
Follow-up 2, mean (SD)	82.19 (16.33)	85.68 (13.37)

Note. Means and standard deviations are based on raw scores that were converted into index scores, which represent the percentage of correct responses.
