# Supplementary material for: Improving adolescents’ knowledge about mental health and depression: a randomized experimental study of web-based information
Source: Front Digit Health. 2025 Nov 18;7:1640366. doi: 10.3389/fdgth.2025.1640366 (PMC12669095; doi:10.3389/fdgth.2025.1640366)
Supplement: Supplementary file 2 [file Datasheet2.pdf]

Supplementary Material

Sample size calculation

We used the study of Ruble et al. (2013) as reference for our sample size calculation. Ruble et al. (2013) provided adolescents with a three-hour information intervention about depression and found a large effect of knowledge increase in the intervention group compared to a control group that did not receive the intervention ($\eta_p^2 = 0.2$). Using a conservative estimate, we expected a small to moderate effect of knowledge increase in our sample at post-test (Cohen's $f = 0.175$). Taken together, for the effect of knowledge gain from pre- to post-test, with $\alpha = .05$ and $1-\beta = .80$, we would need $n = 34$ participants per group to detect an effect of this size, giving a total sample size of $N = 68$, which we slightly exceeded in the present study. Sample size was calculated using G*Power, version 3.1.9.2.
